# The case report of the impact of coach self-supportive behaviors on the communication effectiveness with depressed athletes: The mediating role of intrinsic motivation

**DOI:** 10.1097/MD.0000000000040639

**Published:** 2024-12-13

**Authors:** Jing Zeng, Kun Liu, Li Qin, Yan Shi

**Affiliations:** a Post-doctoral Workstation, Physical Education College of Shanxi University, Taiyuan, China; b Chengdu Sport University, Chengdu, China; c HaiKou College of Economics, Haikou, China; d Physical Education College of Guizhou Normal University, Guiyang, China; e College of Physical Education, Shanxi University, Taiyuan, China.

**Keywords:** coach autonomy-supportive behaviors, communication effectiveness, depressive moods, intrinsic motivation, mediating effect

## Abstract

**Rationale:**

Coach autonomy-supportive behaviors are a crucial factor in the communication effectiveness with athletes experiencing depressive moods. Therefore, clarifying the relationship between coach autonomy-supportive behaviors and the communication effectiveness with athletes experiencing depressive moods is of significant importance for promoting effective communication between coaches and athletes. This study employs in-depth interviews, questionnaires, and stepwise regression analysis, using purposive sampling to investigate coaches and athletes experiencing depressive moods from various regions, levels, genders, and sports in China. The study examines the impact of coach autonomy-supportive behaviors on their communication effectiveness, as well as the mediating role of intrinsic motivation variables, measuring and testing these variables.

**Patient concerns:**

The participants care about whether the coach’s autonomous support behavior can promote communication between them and the coach.

**Diagnoses:**

The athletes participating in the test have a certain degree of depression.

**Interventions:**

For these athletes, coaches adopt autonomous support behavior in training practice to support their decision-making and meet their psychological needs.

**Outcomes:**

Coach autonomous support behavior has a positive supportive effect on the communication effect between athletes with depressive emotions and coaches.

**Lessons:**

(1) Coach autonomy-supportive behaviors can positively predict the communication effectiveness between coaches and athletes experiencing depressive moods; (2) Intrinsic motivation partially mediates the relationship between coach autonomy-supportive behaviors and communication effectiveness between coaches and athletes experiencing depressive moods. The study concludes that coaches can promote athletes’ intrinsic motivation by supporting and encouraging them to make their own choices and affirming the outcomes of those choices. This approach can alleviate athletes’ depressive moods and foster their willingness to actively communicate and share their feelings with coaches, thereby enhancing communication effectiveness.

## 1. Introduction

Communication refers to the process of exchanging information, expressing emotions, and sharing thoughts between individuals or between individuals and groups through verbal or nonverbal means.^[1]^ Effective communication plays a crucial role in promoting physical and mental health, improving work efficiency and life satisfaction, establishing good interpersonal relationships, and maintaining social cohesion and the harmonious and stable development of society. In recent years, there have been many positive examples in sports training management practice that illustrate how the relationship between coaches and athletes, and even with the team, largely depends on communication. Successful coaches are adept at communication and can effectively interact with different parties in any situation, thereby leading their teams to victory. Conversely, communication breakdowns between athletes and coaches often result in poor athletic performance, coaching changes, or even team disbandment. Notable examples include the Ma family army mutiny, the men’s basketball insubordination incident, and the Beijing Shougang women’s basketball team training boycott, among others. These events have sparked significant controversy and academic attention on the issue of effective communication between coaches and athletes experiencing depressive moods. Coaches have also expressed feeling “powerless” in communicating with athletes who have depressive moods. Therefore, how can coaches effectively communicate with athletes experiencing depressive moods? How can they grasp the timing of communication? What communication skills should be employed? How can the communication effectiveness between coaches and athletes with depressive moods be enhanced? These questions have become important topics for current research.

### 1.1. The relationship between coaches’ autonomy-supportive behaviors and the communication effectiveness with athletes experiencing depressive symptoms

Self-Determination Theory^[2]^ is a macro-theory that explores human motivation and personality.^[3]^ Based on past empirical research, it has gradually evolved into a framework composed of Cognitive Evaluation Theory,^[4]^ Organismic Integration Theory,^[2]^ Causality Orientations Theory,^[5]^ Basic Psychological Needs Theory,^[6]^ and Goal Content Theory.^[2]^

According to self-determination theory, the degree of self-determination in human behavior suggests that individuals need external conditions or factors (e.g., autonomy support to meet basic psychological needs) to internalize external motivation into more positive intrinsic motivation.^[7]^ The theory also emphasizes the interaction between the environment and the individual. Autonomy-supportive behaviors (e.g., providing opportunities for choice and showing a certain level of understanding) are more conducive to generating positive impacts on individuals and have been widely supported by empirical research.^[8]^ For example, when parents support their children’s autonomous choices, allow personal expression and decision-making, and accept their emotions, thoughts, and reactions, it positively influences the children’s development of self-independence.^[9,10]^

Coach need-supportive behavior refers to the positive guidance that coaches provide to athletes during interpersonal interactions, encompassing three dimensions: autonomy support, competence support, and relatedness support.^[11]^ Autonomy support involves granting athletes the right to make their own choices, supporting their ideas, and giving them a sense of agency.^[12]^ Based on this, the research suggests that coaches’ autonomy-supportive behaviors positively influence the communication effectiveness between coaches and athletes experiencing depressive symptoms. Thus, the research hypothesis H1 is proposed: Coaches’ autonomy-supportive behaviors have a significantly positive effect on the communication effectiveness between coaches and athletes experiencing depressive symptoms.

### 1.2. Mediating role of internal motivation

Motivation is considered a complex component of an individual’s psychology and behavior. Situational motivation refers to the motivation an individual experiences while engaged in a current activity. In a previous study by German scholars, it was found that engaging in an interesting activity for the sake of receiving a monetary reward leads to a decrease in subsequent intrinsic motivation for that activity.^[13]^ This indicates that material rewards cannot fundamentally enhance an individual’s motivation for an activity. Subsequently, it was mentioned in self-determination theory and motivation theory that intrinsic motivation is described as engagement in a task driven by interest and enjoyment of the task itself, rather than external rewards or avoidance of responsibility.^[5,14]^

Therefore, in training practices, athletes’ interest and enjoyment in the sport stem from their internal motivation, not from material rewards or other external conditions. Previous research has demonstrated a positive relationship between coaches’ autonomy-supportive behaviors and athletes’ internal motivation. These empirical studies provide evidence for the relationship between autonomy-supportive behaviors and internal motivation.^[15]^ Based on this, the research posits that during training and competition, coaches’ autonomy-supportive behaviors can enhance the internal motivation of athletes experiencing depressive symptoms, thereby improving the communication effectiveness between coaches and these athletes. Consequently, the research hypothesis H2 is proposed: Internal motivation mediates the relationship between coaches’ autonomy-supportive behaviors and communication effectiveness.

**Figure 1.**
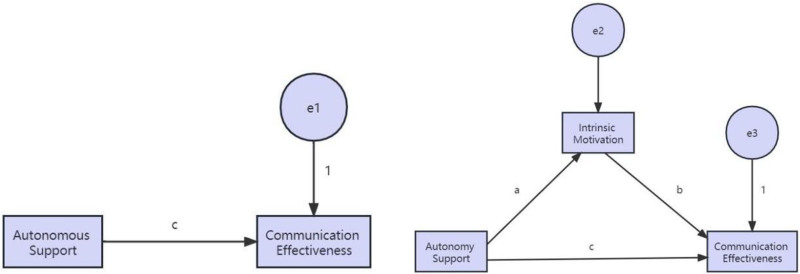
A hypothesis model of the mediation effect of internal motivation in the coaches’ autonomous support behavior on their communication effect.

In the current landscape of psychology, while there have been studies on autonomy-supportive behaviors, motivation, and related topics, much of the focus in research on coaches’ autonomy-supportive behaviors has been on explaining how autonomy-supportive coaching styles can promote athletes’ psychological resilience based on the mediating role of meeting athletes’ basic psychological needs.^[16]^ However, whether coaches’ autonomy-supportive behaviors can create a positive psychological environment for athletes, thus stimulating their internal motivation, and whether athletes’ continuous internal motivation positively affects the communication effectiveness between coaches and athletes, remains unsupported by existing research. Additionally, it is worth further exploring and verifying whether athletes’ internal motivation influences their willingness to engage in proactive communication with coaches and the effectiveness of such communication. This discussion delves into the inherent mechanisms among coaches’ autonomy-supportive behaviors, internal motivation, and communication effectiveness. Drawing upon Self-Determination Theory in psychology and integrating the “shared intention hypothesis” from the field of communication studies, this interdisciplinary study aims to investigate the mediating mechanism of coaches’ autonomy-supportive behaviors in enhancing communication effectiveness between coaches and athletes, with internal motivation as the mediating variable. The goal is to provide theoretical grounds and references for communication effectiveness between coaches and athletes (see Fig. 1).

## 2. Study objects and methods

### 2.1. Study subjects

The study focuses on the impact of autonomy-supportive behaviors in coaches’ communication processes on the effectiveness of communication between coaches and athletes with depressive moods. Coaches and athletes with depressive moods from different regions, levels, genders, and sports were recruited to participate in a questionnaire survey. A total of 1032 questionnaires were distributed and 1032 were collected. After excluding invalid questionnaires due to omissions, multiple selections, or obvious random answers, 881 valid questionnaires remained, resulting in an effective response rate of 85.37%. The athletes and coaches involved in the study are all aware of this study and understand the purpose and significance of each scale test. They agreed to participate in the concurrent table testing of this study. And the patient’s written informed consent has been obtained.

The datasets generated during and analyzed during the current study are not publicly available due the privacy of the coach and athlete, but are available from the corresponding author on reasonable request.

The ethics committee or institutional review committee approved the study by Ethics and Moral Review Committee of Chengdu Sport University.

### 2.2. Patient concerns

The participants care about whether the coach’s autonomous support behavior can promote communication between them and the coach.

### 2.3. Measurement tools

(1) The measurement tool of coaches “independent support behavior is the sub-questionnaire in the coaches” Interpersonal Behavior Questionnaire compiled by Rocchi et al.^[17]^ The questionnaire itself includes three dimensions, both independent support behavior, ability support behavior and relationship support behavior. Based on the needs of this study, 4 items included in “autonomous support behavior” were selected. The scoring was performed by Likert 5 and reliability and validity tests were performed. Testing the internal consistency coefficient, Cronbach α = 0.957, indicates a good reliability of the data obtained by the revised scale.
(2) The internal motivation measurement tool used was the Internal Motivation Scale section of The Situational Motivation Scale.^[18]^ The Intrinsic Motivation Scale is another self-report tool used to assess intrinsic motivation within a situation. This scale aims to evaluate the 4 potential dimensions of intrinsic motivation in specific situations, namely enjoyment of pleasure, perceived ability, importance of effort, and stress and tension. The structural validity of the scale was supported by results consistent with existing theories, and its confirmatory factor analysis was conducted, with a goodness of fit index = 0.80. Using the Likert 5-point scoring method, Cronbach α was measured to be 0.889.
(3) The measurement tool for communication effectiveness used Rebecca B. Rubin’s (1994)^[19]^ Interpersonal Communication Competence Scale developed based on communication studies. The scale clearly indicates that the effectiveness of a goal is determined by whether it has been achieved through communication.^[19]^ Based on the actual needs of this study, 3 items related to communication support were selected, and Cronbach α = 0.777 was measured for this dimension. In addition, in conjunction with Bratman’s “Shared Intention Hypothesis” proposed in 1992, it is mentioned that 2 individuals share a common goal; Two people need to engage in collaborative interaction; Two individuals need to share their psychological states with others during the interaction process, including beliefs, intentions, thoughts, and emotional states. That is to say, when conducting joint actions together, each participant not only needs to have the intention of the activity, but also needs to be aware and consider the intentions and ideas of the other party, and be willing to adjust their own plans to engage in collaborative interaction with the other party, so that participants can take measures together to achieve common goals, rather than attacking each other to achieve their own goals. Based on this, the communication effect between coaches and athletes with depressive emotions in this study will continue to be judged from these three dimensions, and combined with the actual communication between coaches and athletes, a self-developed scale will be developed to measure the communication effect of coaches. Using the Likert 5-point scoring method, Cronbach α was measured to be 0.846.

### 2.4. Mathematical statistics

#### 2.4.1. Structural validity

See Table 1.

**Table 1:** List of spherical results for KMO sample testing and Bartlett.

Sample a sufficient Kaiser–Meyer–Olkin metric	0.740
The sphericity test of the Bartlett	
Approximate chi square	1688.535
Df	171
Sig.	0.000

#### 2.4.2. Content validity

The content validity of the scale represents the degree of agreement^[20,21]^ between the content to be measured and the content actually detected. In this study, the content validity of the initial measurement table was scored by experts (see Table 2), and then the content validity of the initial measurement table was evaluated according to the content validity index (content validity index, CVI), divided into I-CVI (content validity index of item level) and S-CVI (content validity index of scale level).^[21]^ I-CVI is estimated by the random consistency probability and the K* calculation (the calculation announcement is shown below). Finally, after correcting for the random consistency, the larger the I-CVI, the better the content validity of the prompt scale.^[21]^ Since the number of experts scoring the scale is 6, I-CVI, requiring greater than or equal to 0.78.

**Table 2:** Content validity of the initial measurement table.

Clauses and subclauses	Expert score	Number of experts rated as 3 or 4	I-CVI	PC	K*	Evaluate
	A B C D E F					
1	4 3 4 4 4 3	6	1.00	0.016	1.00	Excellent
2	4 4 4 3 4 3	6	1.00	0.016	1.00	Excellent
3	3 3 4 4 3 3	6	1.00	0.016	1.00	Excellent
4	4 4 4 3 3 4	6	1.00	0.016	1.00	Excellent
5	3 4 3 4 3 3	6	1.00	0.016	1.00	Excellent
6	4 4 4 3 3 4	6	1.00	0.016	1.00	Excellent
7	4 3 4 4 3 3	6	1.00	0.016	1.00	Excellent
8	4 3 3 2 3 4	5	0.83	0.094	0.81	Excellent
9	4 3 4 4 3 4	6	1.00	0.016	1.00	Excellent
10	4 3 4 4 3 3	6	1.00	0.016	1.00	Excellent
11	3 3 3 4 3 2	5	0.83	0.094	0.81	Excellent

* *P* < .05.

Step 1: Estimation of the random consistency probability (Pc):


$$\begin{array}{l} Pc=\left[\frac{n!}{A!\left(n-A\right)!}\right]\times 0.5^{n} \end{array}$$

Step 2: Calculation of the K* value:


$$\begin{array}{l} K^{*}=\frac{1-CV1-Pc}{1-Pc} \end{array}$$

CVI is divided into 2 categories: consistent S-CVI (S-CVI/UA, universal agreement), which is the number of entries considered as 3 or 4 points by all experts as a percentage of all entries. Mean S-CVI (S-CVI/Ave), S-CVI/Ave by the mean of all entries I-CVI on the scale.

As can be seen from Table 2, a total of 11 entries had I-CVI > 0.78 and K* > 0.74, indicating excellent content validity of these entries. In the scale, there are 11 items rated as 3 or 4, so the S-CVI/UA of the initial measurement table is 0.818, >0.8, indicating that the content validity index of the scale is good.

Secondly, explore the correlation between independent support, communication effect and internal motivation by the descriptive statistics of mean + SD and related analysis; Thirdly, explore the mediation effect of internal motivation through the multiple gradual regression analysis. Specific steps: the independent variable (X) of this study is “independent support behavior of coaches”; the intermediary variable (M) is “internal motivation”; and the dependent variable (Y) is “communication effect between coaches and athletes experiencing depressive moods.” (1) Make the first regression, Y = cX + e1, and find the c value; (2) Calculate the regression results between the communication effect and the internal motivation of the coach, that is, equation Y = c’X + bM + e3, obtain the values of c’ and b; (3) calculate the regression results between the internal motivation and the independent support behavior of the coach, that is, equation M = aX + e2, and obtain a value. Finally, it was tested by the bootstrap test.

## 3. Outcome

### 3.1. Common method deviation test

Since the data of this study were obtained in the form of questionnaires, a common methodological bias test was required. Exploratory factor analysis was performed using the Harman univariate test, which revealed a total of 4 factors with eigen values >1. The explanation of the first factor is 29.773% variation, which is lower than the critical value of <40%,^[22]^ indicating that there is no significant common methodological bias in this study.

### 3.2. Descriptive statistics and correlation analysis of each study variable

Table 3 presents the mean and standard deviation of the effect of the independent support behavior and the internal motivation, and is tested by the Pearson correlation coefficient between the variables. The results showed that there was a significant positive correlation between the independent support behavior and the communication effect and internal motivation. The significant correlation of the studied variables laid the foundation for subsequent mediation effect testing.

**Table 3:** Study variables describe the statistics and the results of the correlation analysis.

Variable	M	SD	Independent support	Communication effect	Internal motivation
Independent support	4.2909	0.638	1	0.963**	0.958**
Communication effect	4.2914	0.637	0.963**	1	0.935**
Internal motivation	4.2721	0.648	0.958**	0.935**	1

*P < .05.

** *P* < .01.

### 3.3. Test of the mediation effect of internal motivation

According to the aforementioned research hypothesis and the results of the above related analysis, the mediation effect verification method^[23]^ was analyzed by gradual regression (as shown in Table 4): the independent variable (X) of this study is “independent support behavior of coaches”; the mediation variable (M) is “internal motivation”; and the dependent variable (Y) is “Communication effect between coaches and athletes experiencing depressive moods.”

**Table 4:** Regression analysis of the mediating effect model of internal motivation.

Predictive variable	Model 1		Model 2		Model 3	
	β	*t*	β	*t*	β	*t*
Independent support	0.963	106.539***	0.819	26.285***	0.958	99.077***
Internal motivation			0.151	4.850***		
*R* ^2^	0.928		0.930		0.918	
*F*	11,350.480***		5832.433***		9816.271***	

*Note*: Model 1, independent support predicts the communication effectiveness; Model 2, independent support and internal motivation jointly predict the communication effectiveness; Model 3, that is, the autonomous support to predict the internal motivation.

* *P* < .05.

** *P* < .01.

*** *P* < .001.

**Figure 2.**
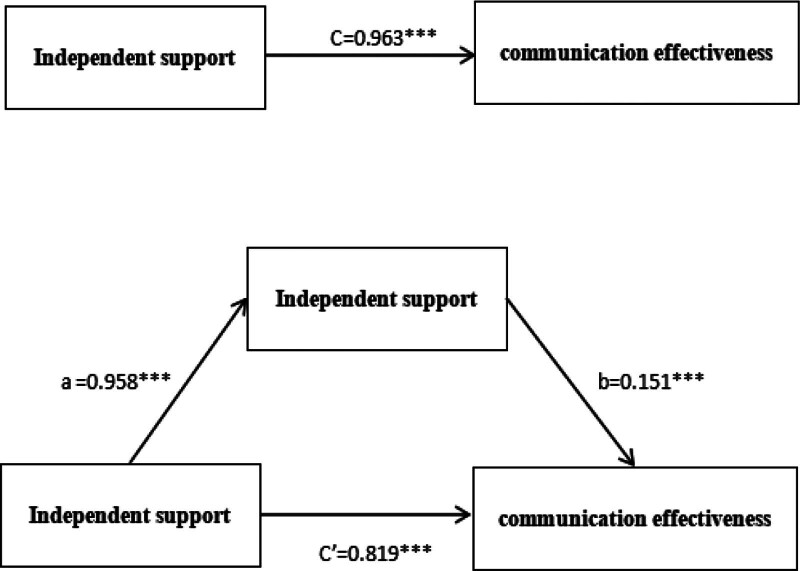
Visual diagram of the path coefficient.

(1) The first regression, Y = cX + e1, and find the c value, that is, the linear regression result of the communication effect and the coaches’ independent support behavior: F = 11,350.480, *P* = .000, indicating that the regression model is meaningful. A *t* test, *t* = 106.539, *P* = .000, the regression coefficient was significant. The normalized regression coefficient was 0.963, namely, in Y = cX + e1, and C = 0.963.

(2) Calculate the regression results of communication effectiveness with internal motivation and coach’s autonomous support behavior, using equation Y = c’X + bM + e3, and obtain the values of c’ and b, which are the regression results of communication effectiveness with internal motivation and coach’s autonomous support behavior, F = 5832.433, *P* = .000. Explain that the regression model is meaningful; The *t* test shows that the regression coefficient between internal motivation and coach’s autonomous support behavior is significant, with *t* (autonomous support behavior) = 26.285, *P* = .000; *t* (internal motivation) = 4.850, *P* = .000; all *P*-values are <.05, indicating that the regression coefficient is significant, resulting in C’ = 0.819, b = 0.151.

(3) Calculate the regression results of internal motivation and coaches’ autonomous support behavior, that is, equation M = aX + e2, and obtain a value, that is, the regression results, F = 9816.271, *P* = .000, indicating that the regression model is meaningful. *t* test of behavioral regression coefficient, *t* = 99.077, *P* = .000, meaningful regression coefficient, coefficient a = 0.958 in equation (see Fig. 2).

### 3.4. Analysis of the bootstrap-test results

Stepwise regression observes the existence of mediating effects by sequentially testing the significance of path coefficients without directly testing the significance of the mediating effects themselves.^[24,25]^ Therefore, this study employed the Process macro program model^[26]^ and utilized bootstrap methods to confirm the mediating effect, as shown in Table 5. Table 5 shows that the total effect of X and Y is 0.9784, with a 95% CI of 0.9604 to 0.9965 and *P* = .000 < .05. This indicates that the total effect of X–Y is statistically significant, meaning that coaches’ autonomous support behavior significantly impacts communication effectiveness. The direct effect of X on Y is 0.8315, with a 95% CI of 0.7694 to 0.8935 and *P* = .000 < .05, confirming a direct effect, that is, coaches’ autonomous support behavior directly affects communication effectiveness. The indirect effect of X on Y is 0.1470, with a 95% CI of ‐0.0381 to 0.4128. Thus, the model in this study, where internal motivation mediates the impact of coaches’ autonomous support behavior on the communication effectiveness between coaches and athletes with depressive emotions, is an indirect mediation model. Additionally, the study performed 5000 bootstrap resamplings with a 95% confidence interval.

**Table 5:** Bootstrap-test of mediation effects of internal motivation.

	Effect value	Boot CI lower limit	Boot CI upper limit	*P*
Total effect	0.9784	0.9604	0.9965	.000
Direct effect	0.8315	0.7694	0.8935	.000
Indirect effect	0.1470	‐0.0381	0.4128	–

Number of bootstrap samples for percentile bootstrap confidence intervals: 5000.

## 4. Conclusion

This study, grounded in self-determination theory and the “shared intention hypothesis” from communication studies, systematically examines the relationship and mechanisms of coaches’ autonomous support behaviors on the effectiveness of coach-athlete communication. It elucidates how coaches’ autonomous support behaviors influence the communication outcomes with athletes experiencing depressive emotions, specifically, the mediating role of internal motivation. The findings offer significant implications and practical value for effective communication between coaches and athletes with depressive moods.

### 4.1. Influence of the coaches’ independent support behaviors on the communication effect

Previous research on coach behavior has mostly focused on the level of coach leadership behavior, such as the impact of coach leadership behavior on athlete engagement, performance, and training satisfaction. However, coach leadership behavior can be divided into democratic and authoritarian types,^[27]^ and coach autonomous support behavior belongs to one type of democratic leadership behavior. Under coach’s autonomous support behavior, coaches tend to focus on athletes, encouraging them to make independent choices and supporting them in making choices. Therefore, the relationship between coaches and athletes belongs to a state of “mutual tolerance” and “mutual prosperity,” which also conforms to the three dimensions proposed by the “shared intention hypothesis” in communication studies, namely (1) two people have a common goal; (2) two people need to engage in collaborative interaction; (3) two individuals need to share their psychological states with others during the interaction process, including beliefs, intentions, thoughts, and emotional states. Currently, there is no in-depth analysis on the communication issues between coaches and athletes with depressive emotions, and there is no discussion on whether there are mediating variables involved. This study cites a measurement scale developed by communication studies on communication effectiveness, and uses coach’s autonomous support behavior as the independent variable. The regression coefficient is 0.963, which has a significant predictive effect on coach communication effectiveness. This is consistent with the statement in the self-determination theory that the closer behavioral regulation is to internal motivation, the stronger a person’s active participation and engagement will be, as well as the positive correlation between self-regulation and psychological needs satisfaction mentioned in previous studies.^[28–30]^ In addition, the process of sports training is actually a combination of the coach’s “teaching” and the athlete’s “practice.” In this process, “communication” is the main medium for achieving the practice of “teaching” and “practicing” in sports training. The efficiency and smoothness of communication have a significant impact on athletes achieving excellent sports results. But the prerequisite for efficient and smooth communication is the attitude of coaches and athletes towards the behavior of “communication” itself. Therefore, coach’s autonomous support behavior has a significant impact on communication effectiveness and has been validated in this study.

### 4.2. Analysis of the mediation role of internal motivation

In terms of mediating effects, this study conducted a stepwise regression analysis with internal motivation as the mediating variable and coach communication effectiveness as the dependent variable. The results showed that internal motivation partially mediates the relationship between coach’s autonomous support behavior and communication effectiveness, thus verifying research hypothesis H2. According to self-determination theory, internal motivation is closely related to 3 psychological needs: autonomy, ability, and association. Mobilizing the internal motivation of athletes with depressive emotions will subsequently affect a certain behavior of individual athletes, which should also include the behavior of athletes actively communicating with coaches. Previous studies have supported the hypothesis that motivation effectively predicts exercise persistence and behavior. For example, a stepwise regression analysis with exercise motivation and exercise atmosphere as independent variables and exercise persistence as dependent variable showed that outdoor exercise atmosphere in adolescents partially mediates the prediction of exercise motivation on exercise persistence.^[14]^ However, in the study of internal motivation in communication behavior, a study has found that coach interpersonal behavior that coach autonomous support behavior positively predicts the basic psychological needs of athletes, thereby affecting their psychological resilience.^[31]^ We did not further explore whether the positive prediction of basic psychological needs of athletes further promotes the daily communication effect between coaches and athletes with depressive emotions. Does internal motivation have a mediating effect among them? Is it a complete intermediary or a partial intermediary? In this study, from the perspective of effect performance, the indirect benefit a = 0.958; b = 0.151; c’=0.819 are both significant, predicting that coach’s autonomous support behavior has a partial mediating effect on communication effectiveness.

## 5. Limitations of the research

This study verified the mediating role of internal motivation in the relationship between coach’s autonomous support behavior and communication effectiveness, and it belongs to a partial mediating role. Based on self-determination theory, through reviewing and analyzing previous research literature, it was found that internal motivation is closely related to 3 psychological needs: autonomy, ability, and association. However, research on communication between coaches and athletes with depressive emotions is relatively weak. However, “communication” is the basic medium for coaches and athletes to implement sports training activities, so further research can be strengthened. Due to the fact that “communication” is not limited to the field of psychology, but also involves related disciplines such as communication, it is hoped that future research can further analyze whether athlete gender can serve as a moderating variable to affect communication effectiveness under coach’s autonomous support behavior, based on the combination of communication theory and measurement scales for the first time in this study, in order to further explore its effects.

## Author contributions

**Conceptualization:** Yan Shi.

**Data curation:** Jing Zeng.

**Formal analysis:** Jing Zeng.

**Funding acquisition:** Jing Zeng.

**Project administration:** Yan Shi.

**Software:** Li Qin.

**Supervision:** Kun Liu, Yan Shi.

**Validation:** Jing Zeng.

**Visualization:** Jing Zeng.

**Writing – original draft:** Jing Zeng.

**Writing – review & editing:** Jing Zeng.
